# Measurement properties of asthma-specific quality-of-life measures: protocol for a systematic review

**DOI:** 10.1186/2046-4053-3-83

**Published:** 2014-07-24

**Authors:** Christian Apfelbacher, Priya Paudyal, Alpaslan Bülbül, Helen Smith

**Affiliations:** 1 Medical Sociology, Institute of Epidemiology and Preventive Medicine, University of Regensburg, Franz-Josef-Strauß-Allee 11, Regensburg 93051, Germany; 2 Division of Public Health and Primary Care, Brighton and Sussex Medical School, Falmer, Brighton BN1 9RD, UK

**Keywords:** Asthma, Health-related quality of life, Validity, Reliability, Responsiveness to change

## Abstract

**Background:**

Asthma is a frequent chronic inflammatory disease of the airways, and the assessment of health-related quality of life (HrQoL) is important in both research and routine care. Various asthma-specific measures of HrQoL exist but there is uncertainty which measures are best suited for use in research and routine care. Therefore, the aim of the proposed research is a comprehensive systematic assessment of the measurement properties of the existing measures that were developed to measure asthma-specific quality of life.

**Methods/design:**

This study is a systematic review of the measurement properties of asthma-specific measures of health-related quality of life. PubMed and Embase will be searched using a selection of relevant search terms. Eligible studies will be primary empirical studies evaluating, describing or comparing measurement properties of asthma-specific HRQL tools. Eligibility assessment and data abstraction will be performed independently by two reviewers. Evidence tables will be generated for study characteristics, instrument characteristics, measurement properties and interpretability. The quality of the measurement properties will be assessed using predefined criteria. Methodological quality of studies will be assessed using the COnsensus-based Standards for the selection of health Measurement INstruments (COSMIN) checklist. A best evidence synthesis will be undertaken if more than one study have investigated a particular measurement property.

**Discussion:**

The proposed systematic review will produce a comprehensive assessment of measurement properties of existing measures of asthma-specific health-related quality of life. We also aim to derive recommendations in order to help researchers and practitioners alike in the choice of instrument.

**Trial registration:**

PROSPERO registration number: CRD42014010491.

## Background

Asthma is a chronic inflammatory disease with variable airway obstruction, characterized by episodes of coughing, wheezing, breathlessness and chest tightness. It is estimated that around 300 million people worldwide suffer from asthma and that globally asthma accounts for about one in every 250 deaths [1]. By 2025, there may be 100 million more people with asthma. The number of disability-adjusted life years (DALYs) lost to asthma is comparable to the DALYs lost for diabetes, cirrhosis of the liver or schizophrenia.

Various medical and non-medical interventions exist to alleviate asthma, many of which have been assessed in randomized controlled trials. Because different outcome measures of various domains (such as disease severity, quality of life, symptoms) are used in different trials, the findings from these trials are difficult to compare. The lack of standardized asthma outcome measurement currently makes truly evidence-based decision making difficult, if not impossible.

One frequently used outcome is ‘health-related quality of life’ (HRQoL) which is a patient-reported outcome (PRO). According to the definition of the U.S. Food and Drug Administration (FDA), a PRO is defined as ‘any report of the status of a patient's health condition that comes directly from the patient, without interpretation of the patient's responses by a physician or anyone else’ [2]. Several reviews of PRO instruments for asthma have been conducted.

A published structured review identified six common asthma-specific quality-of-life measures: the Juniper Asthma Quality of Life Questionnaire (AQLQ-J) [3], the Sydney Asthma Quality of Life Questionnaire (AQLQ-S) [4], the Living with Asthma Questionnaire (LWAQ) [5], the St George's Respiratory Questionnaire (SGRQ) [6,7], the Quality of Life for Respiratory Illness Questionnaire (QOL-RIQ) [8] and the Rhinasthma questionnaire [9]. The measures were reviewed using seven different criteria: conceptual and measurement model, reliability, validity, interpretability, burden, administration format and translations. The review concluded that the instruments differed in almost all criteria, and therefore, it cannot be assumed that they measure the same thing. The authors recommend to select those questionnaires that were designed for asthma and that do not assess symptoms as part of quality of life (QoL). These requirements are fulfilled by the Sydney Asthma QoL Questionnaire (AQLQ-S) [4] and the Living with Asthma Questionnaire (LWAQ) [5]. However, it is also stated that it remains unclear which of the questionnaires best reflects patient perception of QoL.

The Patient-reported Outcome Measurement Group (POMG) in England completed a comprehensive structured review of patient-reported outcome measures for people with asthma and provided recommendations to the Department of Health [10]. The difference to the structured review is that it not only encompasses asthma-specific quality-of-life measures, but also measures of health status, asthma control, utilities and symptoms. Twenty-two asthma-specific measures were evaluated. Based on the volume of evaluations and good measurement and operational characteristics, the original Juniper Asthma Quality of Life Questionnaire [3], the standardized Juniper Asthma Quality of Life Questionnaire [11] and the mini Juniper Asthma Quality of Life Questionnaire [12] as well as the Sydney Asthma Quality of Life Questionnaire [4] were presented to a multidisciplinary panel for discussion. Based on appraisal of evidence by the POMG and taking account of ratings and comments from the panel, the mini AQLQ was recommended as an asthma-specific instrument. Its ease of use and patient acceptability as well as the good concordance between postal and supervised administration was considered to be an important characteristic for measuring outcomes in NHS clinical care.

The third important review comes from the USA. In the light of a lack of adequate outcomes standardization, several National Institutes of Health (NIH) organizations that support asthma research as well as the Agency for Healthcare Research and Quality in the US agreed to a drive towards outcome standardization. As part of this effort, the published documentation relating to asthma-specific quality-of-life measures was reviewed [13]. In the review, the existing instruments were classified as follows:

• *Core outcomes*: selective set of asthma outcomes to be considered by participating NIH institutes and other federal agencies as requirements for institute-/agency-initiated funding of clinical trials and large observational studies in asthma

• *Supplemental outcomes*: asthma outcomes for which standard definitions can or have been developed, methods for measurement can be specified, and validity has been proved but whose inclusion in funded clinical asthma research will be optional

• *Emerging outcomes*: asthma outcomes that have the potential to (1) expand and/or improve current aspects of disease monitoring and (2) improve translation of basic and animal model-based asthma research into clinical research. Emerging outcomes may be new or may have been previously used in asthma clinical research, but they are not yet standardized and require further development and validation

The US review identified 9 instruments for adults, the ABP [14], the Asthma Impact Survey [15], the AQLQ-J-s [11], the mini AQLQ-J [12], the LWAQ [16], the modified AQLQ-S [17], the Asthma Short Form [18], the SGRQ [6,7] and the AQ-20 [17].

None of these qualified as core outcome because they predominantly measured indicators of asthma control (symptoms and/or functional status), failed to provide a distinct, reliable score measuring all key dimensions of the intended construct and/or lacked adequate psychometric data.

All three reviews have failed to perform a systematic literature search and have not included a systematic assessment of the methodological quality of the included studies. The proposed review will therefore systematically assess the measurement properties of asthma-specific quality-of-life measures and include an assessment of the methodological quality of all included studies.

## Methods/design

This study is a systematic review of the measurement properties of asthma-specific measures of health-related quality of life.

### Eligible measures

All measures which are specifically designed to measure health-related quality of life in asthma are eligible.

### Eligible studies

#### *Inclusion criteria*

A study will be included if it is a full text paper, published in English and describes the development (‘inauguration paper’) and/or evaluation of the measurement properties (‘validation paper’) of an asthma-specific measure of HRQoL. The study population should be adults (≥18 years) with asthma. The study should be published as a full text paper and the instrument should be a self-report questionnaire.

Exclusion criteria: Articles reporting on interview instruments or instruments for proxies will not be considered for the purpose of this review. Articles that report an eligible measure, e.g. as an outcome in a clinical trial without any explicit validation will not be considered eligible.

### Literature search

A systematic literature search will be performed in PubMed and EMBASE. Blocks of search terms will be used relating to the following aspects:

• Construct of interest (asthma-specific health-related quality of life): here, the broad search term ‘quality of life’ will be used.

• Target population (adult asthma patients): here, different combinations will be used like ‘allergic’ or ‘atopic’ or ‘bronchial’ and each will be combined with ‘asthma’. The search will be limited to humans.

• Measurement properties: The highly sensitive PubMed search filter for finding studies on measurement properties developed by Terwee et al. will be used to identify relevant articles [19]. This filter has a sensitivity of 97.4% and a precision of 4.4%. A selection of relevant search terms will be used in EMBASE, which has been used before in reviews.

For each of these search strategies, a thorough list of synonyms will be collated using index terms (e.g. MESH terms in PubMed) linked with other free text words. The synonyms will be combined with the conjunction ‘OR’. After that, the searches designed according to the three main aspects will be combined with ‘AND’ in order to get to the list of publications from which the relevant ones will be picked. A further search will be conducted with the names of instruments found in the original search. These names should be combined with AND with the requirements for the target population and measurement properties. The references of all the included relevant articles will also be screened. In addition, websites of relevant professional organizations and institutions will be searched. For all searches, search dates will be provided in the review.

### Study selection

In a first phase, titles and abstracts will be assessed for eligibility. Full text articles will be obtained for the remaining abstracts and again be assessed for eligibility. Each citation will be judged for eligibility independently by two reviewers. Disagreements will be resolved by discussion of all reviewers.

### Data extraction

Two reviewers will independently extract data from each article included. Relevant data from all included articles will be summarized in evidence tables. Evidence tables will contain the following information: characteristics of the instrument (name of measure, domains measured, number of items), characteristics of the study population (geographical location, gender, age, co-morbidities), results for conceptual models and measurement properties (reliability, validity, responsiveness to change according to Table 1) and results for interpretability (including minimal important difference). The evidence tables will be pilot-tested.

**Table 1:** Quality criteria for measurement properties

**Property**	**Rating**	**Quality criteria**
Reliability		
Internal consistency	+	(Sub)scale unidimensional AND Cronbach's alpha(s) ≥0.70
	?	Dimensionality not known OR Cronbach's alpha not determined
	-	(Sub)scale not unidimensional OR Cronbach's alpha(s) <0.70
Measurement error	+	MIC > SDC OR MIC outside the LOA
	?	MIC not defined
	-	MIC ≤ SDC OR MIC equals or inside LOA
Reliability	+	ICC/weighted kappa ≥0.70 OR Pearson's *r* ≥ 0.80
	?	Neither ICC/weighted kappa, nor Pearson's *r* determined
	-	ICC/weighted kappa <0.70 OR Pearson's *r* < 0.80
Validity		
Content validity	+	All items are considered to be relevant for the construct to be measured, for the target population and for the purpose of the measurement AND the questionnaire is considered to be comprehensive
	?	Not enough information available
	-	Not all items are considered to be relevant for the construct to be measured, for the target population and for the purpose of the measurement OR the questionnaire is considered not to be comprehensive
Construct validity		
Structural validity	+	Factors should explain at least 50% of the variance
	?	Explained variance not mentioned
	-	Factors explain <50% of the variance
Hypothesis testing	+	Correlation with an instrument measuring the same construct ≥0.50 OR at least 75% of the results are in accordance with the hypotheses AND correlation with related constructs is higher than with unrelated constructs
	?	Solely correlations determined with unrelated constructs
	-	Correlation with an instrument measuring the same construct <0.50 OR <75% of the results are in accordance with the hypotheses OR correlations with related constructs are lower than with unrelated constructs
Responsiveness		
Responsiveness	+	Correlation with changes on instruments measuring the same construct ≥0.50 OR at least 75% of the results are in accordance with the hypotheses OR AUC ≥0.70 AND correlation with changes in related constructs are higher than with unrelated constructs
	?	Solely correlations determined with unrelated constructs
	-	Correlations with changes on instruments measuring the same construct <0.50 OR <75% of the results are in accordance with the hypotheses OR AUC <0.70 OR correlations with changes in related constructs are lower than with unrelated constructs

Plus sign indicates positive rating; question mark, indeterminate rating and minus sign, negative rating*. MIC* minimal important change, *SDC* smallest detectable change, *LOA* limits of agreement, *ICC* intraclass correlation coefficient, *AUC* area under the curve.

### Assessment of the measurement properties of instruments

The predefined quality criteria for rating the measurement properties of instruments recommended by the COnsensus-based Standards for the selection of health Measurement INstruments (COSMIN) group will be used to assess the measurement properties of the measures [20] (Table 1). These relate to the following measurement properties and aspects of measurement properties: reliability (internal consistency, measurement error, reliability), validity (content validity, structural validity, hypothesis testing) and responsiveness. In addition, we will consider whether the development of any instrument included in the systematic review was based on an a priori conceptual framework/model.

### Assessment of the methodological quality of included studies

The COSMIN checklist [21-23] will be used to evaluate the methodological quality of included studies. In the COSMIN checklist (http://www.cosmin.nl), four domains are distinguished (reliability, validity, responsiveness and interpretability) with related measurement properties and aspects of measurement properties. For each of the measurement properties, the COSMIN checklist consists of 5–18 items covering methodological standards (organized in nine boxes for the nine measurement properties). In addition, each item can be scored on a four-point scale (i.e. ‘poor’ , ‘fair’ , ‘good’ , ‘excellent’). Taking the lowest rating for each item in one box, an overall quality score (poor, fair, good, excellent) is obtained for each measurement property separately.

### Best evidence synthesis

If several studies exist for one measure, findings will be synthesized by combining them, based on number and methodological quality of the studies and consistency of results as previously suggested [24]. The criteria for synthesizing evidence are summarized in Table 2.

**Table 2:** Levels of evidence for the overall quality of a measurement property

**Level**	**Rating**	**Criteria**
Strong	+++ or ---	Consistent findings in multiple studies of good methodological quality OR in one study of excellent methodological quality
Moderate	++ or --	Consistent findings in multiple studies of fair methodological quality OR in one study of good methodological quality
Limited	+ or -	One study of fair methodological quality
Conflicting	+/-	Conflicting findings
Unknown	?	Only studies of poor methological quality

Plus sign indicates positive rating; question mark, indeterminate rating and minus sign, negative rating*.*

## Discussion

The proposed systematic review will produce a comprehensive assessment of measurement properties of existing measures of asthma-specific health-related quality of life. We will highlight the major findings of the review and describe evidence in terms of grade supporting or not supporting the use of any given asthma-specific HRQL tool. We will highlight problems and limitations that we will find across the reviewed tools. The strengths and limitations in the identified evidence (e.g. relating to amount of evidence, validity, feasibility) will be presented and discussed. Finally, we will report about strengths and limitations of our review and highlight future research and policy implications.

We also aim to derive recommendations in order to help researchers and practitioners alike in the choice of instrument. For each instrument identified in the review, a standardized recommendation for usage or required future validation work will be made depending on the best evidence synthesis.

Four categories of recommendation will be made as follows:

a) Outcome measure achieves positive ratings (at least ‘+’) for all measurement properties and is recommended for use.

b) Outcome measure achieves positive ratings (at least ‘+’) for at least two measurement properties, but performance in all other required measurement properties is unclear, so that the outcome measure has the potential to be recommended in the future depending on the results of further validation studies.

c) Outcome measure has low quality in at least one measurement property (at least one ‘-’ rating) and therefore is not recommended to be used any more.

d) Outcome measure has (almost) not been validated. Its performance in all or most relevant measurement properties is unclear, so that it is not recommended to be used until further validation studies clarify its quality.

## Abbreviations

*HrQoL*: Health-related quality of life; *COSMIN*: COnsensus-based Standards for the selection of health Measurement INstruments.

## Competing interests

The authors declare that they have no competing interests.

## Authors’ contributions

CA initiated the protocol, reviewed previous systematic reviews and wrote the manuscript. CA and HES conceptualized the research plan for the proposed systematic review. AB helped with the methodology section. AB, PP and HES reviewed the manuscript for important intellectual content. All authors read and approved the final manuscript.
